# Twenty years trends in mortality rates from stroke in Klaipeda

**DOI:** 10.1002/brb3.499

**Published:** 2016-06-02

**Authors:** Henrikas A. Kazlauskas, Nijole Raskauskiene, Rima Radziuviene, Vinsas Janusonis

**Affiliations:** ^1^ Department of Neurology and RehabilitationKlaipeda University HospitalKlaipedaLithuania; ^2^ Behavioral Medicine Institute Lithuanian University of Health SciencesPalangaLithuania; ^3^ Klaipeda University HospitalKlaipeda UniversityKlaipedaLithuania

**Keywords:** Annual percentage change, joinpoint, mortality, stroke, trends

## Abstract

**Background:**

During the past decades, mortality from stroke decreased in many western European countries; however, changes concerning long‐term stroke mortality in eastern European countries are less evident.

**Objective:**

To assess age‐ and gender‐specific trends in stroke mortality in Klaipeda (Lithuania) from 1994 to 2013.

**Design:**

Descriptive epidemiological study.

**Setting/subjects:**

Permanent population of Klaipeda.

**Methods:**

Data on 2509 permanent residents of Klaipeda aged 35–79 years who died from stroke between 1994 and 2013 were gathered. Directly, age‐standardized (European population) stroke mortality rates were analyzed using joinpoint regression separately for specific age groups (35–64, 65–79, and 35–79 years) and by gender. Annual percentage change (APC) and 95% CIs were presented.

**Results:**

Stroke mortality in the 35‐ to 79‐year‐old age group peaked in 1994–1997, it then decreased by −9.9% (95% CI: −18.7, −0.2) yearly up until 2001 and leveled off by −0.2% (−5.1, 4.9) between 2001 and 2013. Among men aged 35–64 years, mortality decreased substantially by 12.8% (−21.5, −3.3) per year from 1994 to 2001 and turned positive by 6.3% (0.8, 12.1) between 2000 and 2013. Among women aged 35–64 years, mortality decreased significantly by 15.5% (−28.1, −0.7) from 1994 to 2000. There was evidence of recent plateauing of trends for 35‐ to 64‐year‐old women between 2000 and 2013. In the 65‐ to 79‐year‐old age group, mortality decreased from 1994 onward yearly by −5.5% (−7.9, −3.0) in women and by −3.3% (−5.6, −0.9) in men.

**Conclusions:**

Joinpoint regression revealed steadily decreasing trend in stroke mortality between 1994 and 2001. The decline in death rates flattened out in the recent decade. Mortality rates varied among age groups and were more pronounced in adults aged 35–64 years. It is essential to monitor and manage stroke risk factors, especially among middle‐aged population.

## Introduction

In many countries, stroke is the third most common cause of death after heart attack and cancer (Redon et al. 2011); only in United States, it has dropped to fourth place in recent years (Lackland et al. 2014). About 29.6% of all deaths worldwide (15,616.1 million deaths) were caused by cerebrovascular diseases in 2010 (Nichols et al. 2014). Stroke is one of the five most common causes of morbidity in the developed and developing countries (Mirzaei et al. 2012). It has significant socioeconomic consequences for patients, their partners, and society as a whole (Jennum et al. 2015). In many countries, the burden of stroke on the public as well as a large number of deaths from stroke among the elderly is expected to increase (Kunst et al. 2011; Sienkiewicz‐Jarosz et al. 2011). During the last two decades, significantly higher mortality from stroke was observed in central and eastern Europe than in western Europe; however, in the past decade, it also declined in many postcommunist countries (Redon et al. 2011; OECD, 2012; Davídkovová et al. 2013). The total cerebrovascular mortality in the eastern European countries is more than twice as high as in the western Europe. Decline in stroke mortality in eastern Europe began later: a more pronounced decrease occurred after 1998 (annually decreasing by 3.21%). Furthermore, the decline was slower compared to the Western countries (Helis et al. 2011). The epidemiological study conducted in Kaunas as part of the WHO Multinational Monitoring of Trends and determinants in Cardiovascular Diseases (MONICA) project and the scientific analysis of official mortality statistics performed in Klaipeda city (Rastenyte et al. 2006; Kazlauskas et al. 2011) showed significant decrease in stroke mortality in Lithuania over the last two decades. Despite the positive developments in reducing mortality from stroke in our country, it remains one of the highest among the Baltic and central European countries (OECD, 2012). The joinpoint regression model is useful for identifying and describing the occurrence of changes in different time periods throughout trends in data (Kim et al. 2000). The aim of this work is to assess changes in stroke mortality trends in Klaipeda (Lithuania) during the period of 1994–2013 using joinpoint regression models.

## Methods

The study covered permanent population of Klaipeda which decreased from 203,000 in 1994 to 158,000 in 2013. The official mortality statistical data (death certificates, statistical form no. 106/a) were used to evaluate case ascertainment of stroke per 100,000 of Klaipeda population.

We limited our analysis to people aged up to 79 as it approximates Lithuanian life expectancy. In addition, we wanted to compare mortality changes between the working‐age population and the elderly. In our analysis, we focused on the following age groups: 35–64, 65–79, and 35–79 years. The mortality trend data were expressed in the form of rates over time. Data on number of deaths and a corresponding risk population, by sex and in 5‐year age groups (up to 79 years) for each calendar year, were obtained from Klaipeda Civil Registry Office and Klaipeda Data Preparation division of Statistics Lithuania. Direct method was used to perform age adjustment to the European standard population (Waterhouse et al. 1976).

All stroke deaths were classified according to the *International Classification of Disease* codes in use at the time the deaths were reported. ICD‐9 was used to code deaths between 1994 and 1996, whereas ICD‐10 and ICD‐10 AM were applied for the periods 1997–2012 and 2012–2013, respectively. Results were then stratified by gender and presented in combination for hemorrhagic stroke (ICD‐9 430–432, ICD‐10, and ICD‐10 AM I60–I62) and ischemic stroke (ICD‐9 433–436, ICD‐10, and ICD‐10 AM I63–I64).

### Statistical analysis

The trend analysis for age‐adjusted mortality rates (dependent variable) was performed using version 3.0 of Joinpoint software (Surveillance Research Programme of the US National Cancer Institute) (Kim et al. 2000; US National Institutes of Health, 2005). Age‐adjusted mortality rates were calculated in Joinpoint. Excel software package was used to create the input data text file for each analyzed age group (35–64, 65–79, and 35–79 years). The data file contained one by variable (Sex: 0 = male and female, 1 = male, and 2 = female) and the independent variable was Year (1994 through 2013 inclusive). For each Sex/Year combination, there was a complete set of adjustment variable values: an Age variable in 5‐year age groups (35–39 years to 75–79 years), Count (number of deaths, nominator), Population (population at risk, denominator), and Standard Population (European standard population, 1976) (Kim et al. 2000). The standard population appeared on every data record and the value corresponded to the associated age group.

Initially, scatter plots opposing age‐adjusted mortality rates and calendar years were built in order to better visualize the function that might better express the relationship between these variables. Low‐degree polynomial models were expected to fit better to the mortality rate. As a measure of precision, the coefficient of determination (*R* ^2^) was used.

Joinpoint analysis identifies inflexion points (“joinpoints”) at which there is a significant change in the trends, using a series of permutation tests, with Bonferroni adjustment for multiple comparisons. The two‐sided significance level was set at *P* < 0.05 for all tests. The number and location of significant joinpoints by sex and age group (maximum of 3) was determined using a log‐linear model, and the annual percentage change within each segment was calculated. Use of a log‐linear model enables the analysis of constant percentage (rather than absolute) change in prevalence over time. In applying Joinpoint software, the different settings of parameters will lead to differences in the model selection. In addition, the software tests whether the slope of each trend segment differs significantly from the segment immediately preceding it by a *t* test.

Average annual percentage change overall (1994–2013) was calculated with respect to the underlying joinpoint model. The percentage change (PC) in mortality rate 1994–2013 was presented as: (rate at last year of period segment – rate at first year of period segment)/rate at first year of period segment.

The APC was tested to determine whether a difference exists from the null hypothesis of no change (0%). In the final best fitting model, each joinpoint informs a statistically significant change in trends (increase or decrease) and each of those trends is described by an APC (Kim et al. 2000; Yang et al. 2003).

## Results

**Figure 1:**
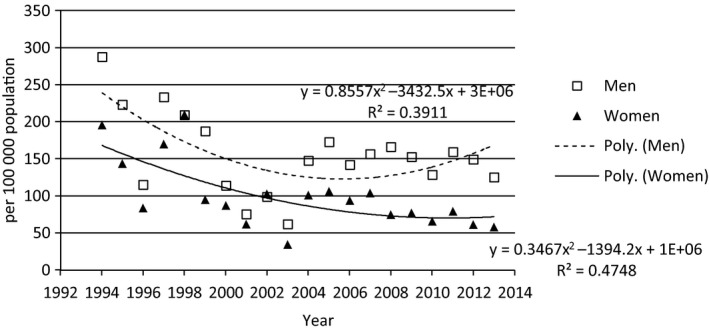
Trends in age‐adjusted mortality rates from stroke among both Klaipeda men and women aged 35–79 years during 1994–2013. Rates are expressed per 100,000 persons and are age adjusted to the European population.

Combined stroke mortality rates decreased substantially in both sexes between 1994 and 2013. Age‐adjusted (European population) stroke mortality in all age groups was higher among men than among women. The overall age‐adjusted stroke mortality rate declined from 1994 to 2013 by 35% in men and by 63% in women (Fig. 1; Table 1).

**Table 1:** Modeled (final model joinpoint regression analysis) death rate and percentage change according to gender and age

Gender and age	Modeled death rate per 100,000		PC^a^ (%)
	1994	2013	1994–2013
All, 35–79	175.3	86.9	−50
Men			
35–64	97.4	74.1	−24
65–79	764.6	405.9	−47
35–79	197.5	128.3	−35
Women			
35–64	48.6	21.1	−56
65–79	686.2	235.2	−66
35–79	164.4	60.5	−63

a PC, percentage change; [(rate at last year of period segment − rate at first year of period segment)/rate at first year of period segment] × 100.

The results for the fitted quadratic model are given below. The quadratic model fits much better than the linear model (for men *R* ^2^ = 0.39; for women *R* ^2^ = 0.48). Plots of the fitted MRs are shown in Figure 1.

### Resulting figures of joinpoint trends for stroke mortality in Klaipeda 1994–2013

To provide a more nuanced examination of the data in each gender and age group, changes in the trend during the 20‐year period were detected by joinpoint regression modeling.

**Figure 2:**
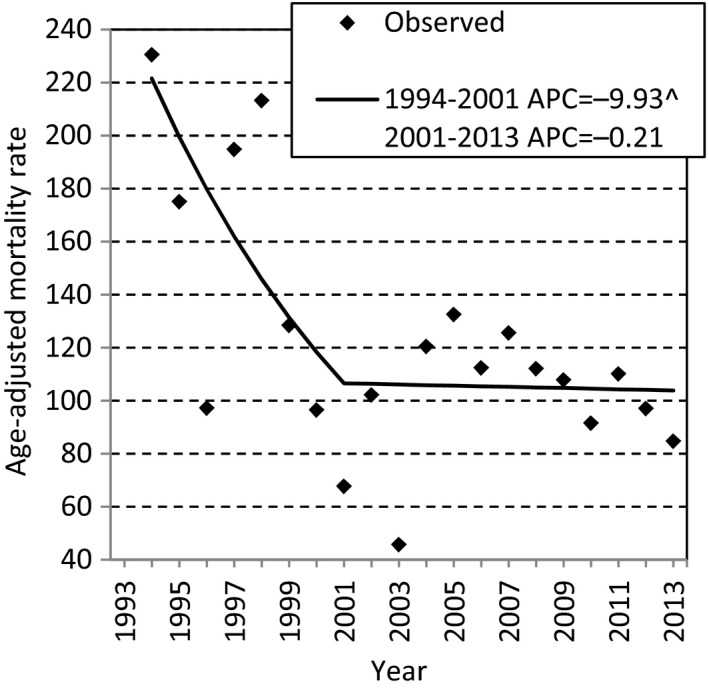
Observed mortality rates and estimated trends from joinpoint analysis among Klaipeda population aged 35–79 years.

Our results indicate that stroke mortality in Klaipeda peaked in the period 1994–1997; it then began to decrease by −9.9% (95% CI: −18.7, −0.2) yearly up until 2001 and leveled off by −0.2% (−5.1, 4.9) between 2001 and 2013 (Fig. 2).

**Figure 3:**
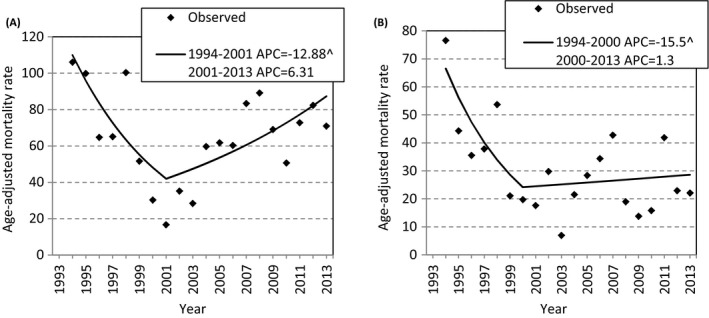
Observed mortality rates and estimated trends from joinpoint analysis among men (A) and women (B) aged 35–64 years.

In best‐fit model, no significant trend was detected for men aged 35–64 years, and the fit of the line is questionable. Best‐fit model with three joinpoints showed that, in the year 2001, the change in trend occurred with a significant decrease followed by a marked increase up until 2005 after which the rates remained stable (data not shown). It should be noted that although a model with additional joinpoints will result in a better fit, a smaller number of joinpoints facilitates interpretation. The two lines seem to fit the data reasonably well. In the model with one joinpoint, among men aged 35–64 years, the mortality rate decreased by 12.8% (−21.5, −3.3) per year from 1994 to 2001 and turned positive by 6.3% (0.8, 12.1) between 2000 and 2013. The joinpoint model seems to fit the data well. Each line has a significant slope (Fig. 3A).

Among women aged 35–64 years, mortality decreased significantly by 15.5% (−28.1, −0.7) from 1994 to 2000. There was evidence of recent plateauing of trends for women in the 35–64 years of age group between 2000 and 2013. Mortality turned positive by 1.2%, albeit with a wide CI that included 0% (−5.7, 8.9) (Fig. 3B).

The age group analysis showed a significant overall mortality decrease by 4.4% (−6.6, −2.2) in the 65‐ to 79‐year‐old age group over the length of the entire study period (1994–2013). Joinpoint analysis does not show the existence of statistically significant points of change in this group.

**Figure 4:**
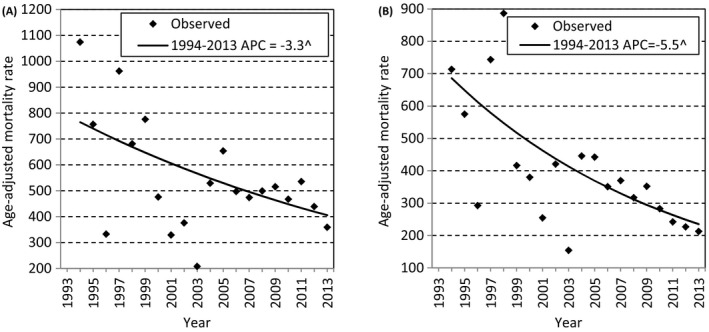
Observed mortality rates and estimated trends from joinpoint analysis among men (A) and women (B) aged 65–79 years.

Even though no joinpoints were identified, the trend over the entire 20 years of analysis was significantly negative in both sexes aged 65–79 years. Among men aged 65–79 years, the mortality rate decreased by 3.3% (−5.6, −0.9) per year from 1994 to 2013 (Fig. 4A), whereas among women aged 65–79 years, it decreased by 5.5% (−7.9, −3.0) per year from 1994 to 2013 (Fig. 4B).

## Discussion

### Summary of findings

The study found that age‐adjusted stroke mortality rates in Klaipeda (Lithuania) declined from 1994 through 2013 by 50% (35% among men and 63% among women aged 35–79 years).

Our results indicate that stroke mortality in Klaipeda population aged 35–79 years peaked in the period 1994–1997; it began to decrease after that by −9.9% per year up until 2001 and leveled off between 2001 and 2013. Among men aged 35–64 years, the mortality rate decreased substantially by 12.8% per year from 1994 to 2001 and turned positive by 6.3% between 2000 and 2013. Among women aged 35–64 years, mortality decreased significantly by 15.5% from 1994 to 2000. There was evidence of recent plateauing of trends for women in the 35–64 years age group between 2000 and 2013. Stroke mortality rates declined incessantly between 1994 and 2013 among both sexes aged 65–79 years.

### Comparison with other studies

In all western European countries, except Greece, steady decreasing stroke mortality trends were observed as from 1985 (Levi et al. 2009). Similarly to the results of our study, stroke mortality rates in all EU Member States fell since 1995 but, in contrast to our study, with a more pronounced decrease after 2001 (OECD, 2012). Our data show a striking decline in the overall male–female stroke mortality up until 2001 which then leveled off by 2013. Vaartjes et al. (2013) used a joinpoint regression analysis to show slowly decreasing ischemic stroke death rates up until 2000 and a remarkable decline in stroke mortality after 2000 in all age–sex groups, except for young men, similarly as in our study. U.S. authors (study period of 1969–2013) undertook joinpoint analysis of U.S. National Vital Statistics data which revealed the decreasing trend in stroke mortality corresponding to the last segment of our study: a decrease from 5.5% during 2001–2007 to 3.0% during 2007–2013 (Ma et al. 2015). The study conducted by Bennett et al. (2013) in the Republic of Ireland and Northern Ireland when using joinpoint regression analysis showed, in contrast to our study, a significant slowdown in the rate of change in stroke mortality from 2005 in both sexes.

Changes in stroke mortality at a younger age are difficult to compare due to the variety of the age groups analyzed in the literature. In the European Union countries, annual mortality decline in the period 1980–2007 in the 35–44 years age group was −2.5% in both sexes. Steeper decline in cerebrovascular mortality rate in this age group occurred only in the mid‐late 1990s (Bertuccio et al. 2011). Such results were confirmed by our study: in the period 1994–2001, male and female rates of mortality decreased significantly (−12.8% per/year and −15.5% per/year, respectively), albeit our age group was older. Moreover, among men aged 35–64 years, the APC mortality rate turned positive by 6.3% (0.8, 12.1) between 2000 and 2013. Our analysis showed that the mortality rate for women aged 35–65 and 65–79 years and for men aged 65–79 years was the lowest in 2003. The causes of these findings remain unclear. We can only speculate about the phenomenon of sharp changes in stroke mortality as corresponding to possible changes in mortality known as “epidemic patterns” common in this period of time to most post‐Soviet and eastern European countries (Mirzaei et al. 2012). The authors of the recent large European survey reported evidence demonstrating that decrease in mortality rates may have begun to slow down or even reverse in some specific subpopulations in the past decade (Nichols et al. 2014). A relative increase in middle‐aged male mortality from stroke in a similar period of time (1979–2005), corresponding to our survey data, was found in the study of Sutton et al. (2010). Female mortality rates in our study decreased quite evenly each year without more distinct fluctuations. In the group of 65–79 years, joinpoint analysis showed substantial decrease in mortality during the whole study period: APC −3.3 per/year in men and −5.5 per/year in women. Compared with the younger age group, the mortality rate decline in the older group was several times lower in the first period of the survey. Different results were obtained by Australasian researchers who applied joinpoint Poison regression model to analyze mortality rate in the 65–74 years age group. Their study found no significant decrease in the periods 1980–2005 and 1996–2005: 5.3% per/year (−5.6, 5.0) in women and −6.6% per/year (−7.6, 5.7) in men in two periods, respectively (Backholer et al. 2011). Despite these findings, Swedish authors observed average annual male and female mortality decline changes (−4% per year) based on national registry data, which are similar to our study results. Their analysis covered a similar period of time, even though the range of age was wider (60–85 years old) (Modig et al. 2013).

### Factors influencing stroke mortality

Many authors agree that decline in early case fatality plays a very important role in reducing mortality from stroke. The WHO MONICA study found that in the 35–65 years age group, mortality decreased, probably due to reduced case fatality and not because of the reduced number of new stroke cases (Sarti et al. 2003). After having explored stroke mortality in seven western European countries, Kunst et al. (2011) reported a strong decline in stroke mortality which is likely to be attributable to declines in case fatality and in incidence rates. Moreover, the researchers argue that reduced case fatality shows an improved management of stroke risk factors, and consequently, possibly less severe stroke events (Modig et al. 2013), better diagnostics, better treatment of patients in the acute stage, as well as increased funding for this area of medicine (Redon et al. 2011). The present study supports these considerations; case fatality in the acute stage of stroke began to decline, which may be the result of the organizational improvements of the acute stroke care during the observational period. First, an educational program designed for city residents, ambulance personnel, and family physicians was launched. Second, an acute stroke unit was established at Klaipeda city hospital in 1994 offering intensive monitoring and nursing for a short period of time, including early thrombolytic treatment. After their discharge, patients could benefit from therapy in neurological rehabilitation centers (Teasell et al. 2014).

Recent large‐scale studies conducted in Western countries have confirmed that the decrease in mortality from stroke, during the past decades, was mostly influenced by reduced morbidity and case fatality from this disease (Sienkiewicz‐Jarosz et al. 2011; Lackland et al. 2014). When analyzing controllable factors possibly influencing the decline in stroke mortality in Klaipeda city, these have to be assessed in the context of the whole country, since stroke risk factors in Klaipeda city have not been studied in recent years. The exclusive feature specific for the inhabitants of Klaipeda lies in the fact that the city is close to the sea and frequent changes of diverse meteorological factors could have significantly affected the hemodynamics of the city's population (Zhang et al. 2009; Aubinière‐Robb et al. 2013), as compared to those living further away from the sea.

Recent studies on cardiovascular mortality trends showed that decreasing death rate is largely attributable to improvements in control of hypertension, hyperlipidemia, and smoking cessation (Ma et al. 2015). The analysis of risk factors conducted in 1983–2002 in Lithuania showed a statistically significant decrease in the average systolic blood pressure in the 35–64 years age group for men (from 138.8 to 136.4 mmHg) and women (139.7 to 133.2 mmHg) as well as in the diastolic arterial blood pressure only for women (from 86.5 to 82.7 mm Hg). Some studies have found increasing obesity rates in young and middle‐aged people, which may have negative impact on cardiovascular disease risk (Rosengren et al. 2013). Furthermore, cardiovascular diseases were shown to be most common causes of death among younger ischemic stroke survivors (Giang et al. 2013). During the first decade of our investigation (1983–2003), a statistically valid decrease in body mass index in Lithuanian men was observed (Nichols et al. 2013). Some authors associate the attenuation in declining mortality rate for obesity‐related disease (heart disease, stroke, and diabetes) with increased prevalence of obesity in today's society (Ma et al. 2015). Unfortunately, we do not have data on changes in obesity in Lithuania in the second decade of our study, when the stroke death rate leveled off in the middle‐aged group. There is, however, evidence of an increasing diabetes prevalence trend in Lithuania among adults as well as of high rates of NCDs risk factors overall (Nichols et al. 2013).

Smoking is one of strongest stroke risk factors and stroke survivors who smoke have an increased risk of all‐cause mortality (Levine et al. 2014). Generally, men smoke twice as often as women (World Health Organization, 2013). During the investigation period, change rates in smoking behavior were different among men and women. In 2008, there were 38.8% of adult male and 14.9% of female smokers. From 1994 to 2008, smoking rates among women have increased twice, while among men the increasing rates in the period of 1994–2000 started to decline later on until they reached the 1994 year level in 2008. The number of those who stopped smoking increased among both genders (Dambrauskiene et al. 2010). These changes among smokers may be associated with the efficient domestic tobacco control policy (Kalediene and Sauliune 2013). European cardiovascular disease statistics show that Lithuania is one of the countries where there is a wide use of strong alcoholic beverages. Lithuanian researchers found that, in 2006, 30% of men and 10% of women in our country used strong alcoholic beverages at least once a week (Grabauskas et al. 2007). During our study period, bad eating habits were also more prevalent among men and among less educated persons (Grabauskas et al. 2004). These aforementioned male–female characteristics and differences in habits in our country may have influenced higher male mortality rates in our study. New research shows that, over the past two decades, the dietary habits of Lithuania's inhabitants have improved. The percentage of energy from saturated fatty acids in aged (25–64 years) people of rural population has decreased from 18.0 to 15.1 among men and from 17.6 to 14.8 among women. Favorable trends in fatty acids composition were caused by increased use of vegetable oil for cooking and the replacement of butter spread with margarine. Since 1987, the mean value of total cholesterol has decreased by 0.6 mmol/L. Total dietary decline in serum cholesterol was 0.26 mmol/L (43.3%) among men and 0.31 mmol/L (50.8%) among women (Ramazauskiene et al. 2011). Overall, slowdown in the decline of stroke mortality, especially in the younger age group in the second decade of our survey, can be partly explained by diminishing returns on previously successful interventions as well as by lack of effective management of risk factors (Backholer et al. 2011).

### Stroke mortality and gender

Our research showed that mortality among men was higher as compared to women in all age groups. A recent large‐scale study on time trends in cardiovascular and all‐cause mortality revealed similar results: it found higher mortality rates in eastern European men than in women (Helis et al. 2011). Another important observation is related to the difference in mortality rate among sexes over the distinct periods of our study: in the first study period (1994–2000), significantly reduced mortality for both sexes was observed, whereas in the second period (2000–2013), a significant increase in APC death rate by 6.3% (0.8, 12.1) in young males only and a plateau (APC +1.3; *P* = 0.7; 2000–2013) in trends among females aged 35–64 years was found. Higher death rate from stroke among men is confirmed by data from epidemiological studies, although the results of systematic literature reviews show that stroke events are more severe among women and their 28‐ to 31‐day mortality is 1.25 times higher compared to men (Appelros et al. 2009). The authors explain this by referring to higher comorbidity rates, more frequent stroke risk factors (arterial hypertension, atrial fibrillation, anemia, heart failure) and higher frequency of prestroke disability in females. Mortality rates are higher among men in some studies, while long‐term ADL dependency seems to be more common among women. In addition, women develop stroke later in life and they live longer. Women have less favorable functional outcome because of higher age at stroke onset and more severe strokes (Appelros et al. 2009). Some authors propose a hypothesis that the progressive male hormones (testosterone) deficiency is a major cause of difference in survival between the sexes in patients with stroke (Giang et al. 2013). Moreover, our study found that male standardized mortality rate during the study period decreased by 35% and that of females by 63%. One can only speculate why these mortality rates for men during the whole study period dropped by almost half as much as those for women. In their study, Levi et al. (2009) reported proportionally greater recent cardiovascular and cerebrovascular stroke mortality declines in men than in women in most countries which are attributable to different trends in tobacco consumption in both sexes. An overview of the risk factors of ischemic stroke in young adults in the last years revealed rising prevalence of “traditional” vascular risk factors in young age groups (Maailjwee et al. 2014). Recent research has shown that heavy consumption of alcohol increases the possibility of dying from stroke (Rantakömi et al. 2014). Bearing in mind that alcohol use (Dambrauskiene et al. 2010; Mirzaei et al. 2012; Kalediene and Sauliune 2013) and smoking (Dambrauskiene et al. 2010) are more prevalent among men, especially at a young and middle age, as compared to women in Lithuania, and the fact that men, for various reasons, consume less fruits and vegetables (OECD, 2012), one may think that these factors may have influenced the smaller decrease in mortality in young males in the first study period and, moreover, positive mortality trends in the second period of the study.

At the beginning of the 21st century, the mortality rate from circulatory system diseases for men and women in Lithuania ranged remaining high. For instant, in 2007–2009, mortality from these causes has remained three times higher than in the “old” 15 EU countries (Stankuniene and Jasilionis 2011). In spite of these high mortality rates, research conducted in Lithuania showed that, during our study period, healthy life expectancy, which is an important indicator for health policy development in Lithuania, was increasing (Kalediene and Petrauskiene 2004). Besides, mortality from leading causes of death (Kalediene and Sauliune 2013) and mortality from cardiovascular diseases in 2001–2010 decreased by 11.5% in each of the gender group (Rinkūniene et al. 2013).

In addition to the generally accepted behavioral factors which influence mortality from stroke, there may be other reasons, such as country's historical background, socioeconomic situation, conditions related to living environment, as well as cultural and health policy (Bajaj et al. 2010; Mirzaei et al. 2012; Redon et al. 2011). These factors have possibly positively influenced stroke mortality decline in Klaipeda population during the study observational period. It remains vitally important to monitor and work toward reducing preventable risk factors for stroke, especially for younger age groups. “There may still be time for public health policy and action to have an impact on these risk factors to prevent such impacts” (Nichols et al. 2013).

### Strengths and limitations

Mortality data abstracted from death certificates provide an important source of information to measure the burden of disease in a population, and official mortality registers are often used in epidemiologic research (Kelsh et al. 2009). However, analysis of death certificates might lead to misclassification. Despite the doubts about the validity of data collected from this source, “the statistical update is valuable resource for researchers, clinicians, healthcare policy makers” (Rosamond et al. 2008). As regards the availability and usefulness of this data source, the weak point is that part of stroke patients die at home and their diagnosis is not confirmed by routine radiological examination. In addition, higher validity of data may have been influenced by improved hospitalization of patients having this profile in Klaipeda city. Since the end of 1995, main organizational principles of the Helsingborg declaration on stroke management strategy in Europe, adopted in the same year on November 8–9, began to be actively implemented in Klaipeda; therefore, the majority of patients with acute stroke were admitted to hospital and subjected to the routine diagnostic (brain computed tomography) examination. In the period of 1994–2013, out of all patients included in the study in Klaipeda city, only 11.8% died at home. Taking this into account, one can guess that patients in our study were accurately diagnosed with stroke with little margin of error. In the study results, we did not provide data on the specificities related to stroke types; therefore, we did not analyze peculiarities of mortality rates in these patient groups. As one of the study advantages, one could refer to a rather broad range of age and a long death observation period which allows obtaining a complete picture of changes in mortality from stroke in Klaipeda city.

## Conclusions

Joinpoint regression revealed steadily decreasing trend in stroke mortality between 1994 and 2001. The decline in death rates flattened out in the recent decade. Mortality rates varied among age groups and were more pronounced in adults aged 35–64 years. It is essential to monitor and manage stroke risk factors, especially among middle‐aged population.

## Conflict of Interest

None declared.
